# Bone Sparing Effect of a Novel Phytoestrogen Diarylheptanoid from *Curcuma comosa* Roxb. in Ovariectomized Rats

**DOI:** 10.1371/journal.pone.0078739

**Published:** 2013-11-11

**Authors:** Duangrat Tantikanlayaporn, Patsorn Wichit, Jittima Weerachayaphorn, Arthit Chairoungdua, Aporn Chuncharunee, Apichart Suksamrarn, Pawinee Piyachaturawat

**Affiliations:** 1 Department of Physiology, Faculty of Science, Mahidol University, Bangkok, Thailand; 2 Department of Anatomy, Faculty of Medicine, Siriraj Hospital, Mahidol University, Bangkok, Thailand; 3 Department of Chemistry, Faculty of Science, Ramkhamhaeng University, Bangkok, Thailand; Oklahoma State University, United States of America

## Abstract

Phytoestrogens have been implicated in the prevention of bone loss in postmenopausal osteoporosis. Recently, an active phytoestrogen from *Curcuma comosa* Roxb, diarylheptanoid (DPHD), (3*R*)-1,7-diphenyl-(4*E*,6*E*)-4,6-heptadien-3-ol, was found to strongly promote human osteoblast function *in vitro*. In the present study, we demonstrated the protective effect of DPHD on ovariectomy-induced bone loss (OVX) in adult female Sprague-Dawley rats with 17β-estradiol (E_2_, 10 µg/kg Bw) as a positive control. Treatment of OVX animals with DPHD at 25, 50, and 100 mg/kg Bw for 12 weeks markedly increased bone mineral density (BMD) of tibial metaphysis as measured by peripheral Quantitative Computed Tomography (pQCT). Histomorphometric analysis of bone structure indicated that DPHD treatment retarded the ovariectomy-induced deterioration of bone microstructure. Ovariectomy resulted in a marked decrease in trabecular bone volume, number and thickness and these changes were inhibited by DPHD treatment, similar to that seen with E_2_. Moreover, DPHD decreased markers of bone turnover, including osteocalcin and tartrate resistant acid phosphatase (TRAP) activity. These results suggest that DPHD has a bone sparing effect in ovariectomy-induced trabecular bone loss and prevents deterioration of bone microarchitecture by suppressing the rate of bone turnover. Therefore, DPHD appears to be a promising candidate for preserving bone mass and structure in the estrogen deficient women with a potential role in reducing postmenopausal osteoporosis.

## Introduction

Osteoporosis is a serious worldwide health problem that primarily effect middle-aged and elderly women [1], [2]. It is characterized by reduced bone mass and the deterioration of bone microarchitecture leading to increase the risk of bone fragility and fracture [3]. An accelerated rate of bone resorption in menopausal and post-menopausal women is associated with reduced levels of the hormone estrogen [4]. Recently, efforts to reduce bone loss in menopausal osteoporosis have been focused on compounds with the potential to preserve bone mass through inhibition of osteoclastic bone resorption or stimulation bone formation [5]. Among therapeutic agents, estrogen is the most effective compound and is capable of limiting bone loss and reducing the rate of bone fractures in postmenopausal women [6], [7]. However, long-term treatment with estrogen is limited due to its carcinogenic risk and feminizing effects.

Phytoestrogens, non-steroidal plant-derived compounds with estrogenic activity, have received increased interest as estrogen alternatives to alleviate bone loss. Studies have suggested that a diet rich in phytoestrogen may relieve menopausal symptoms and protect against estrogen-associated diseases, including breast cancers, cardiovascular diseases, and osteoporosis [8], [9]. Isoflavones, such as genistein and daidzein the major phytoestrogens in soybeans, are the most extensively studies phytoestrogens. These compounds inhibit osteoclast bone resorption and suppress osteoclast activity and survival *in vitro* [10], [11]. In addition, isoflavones have been identified as naturally occurring selective estrogen receptor modulators (SERMs) and as bone-sparing agents [12], [13]. The known properties of phytoestrogens suggest that these compounds may be alternatives to estrogen for preventing and treating osteoporosis in postmenopausal women.

*Curcuma comosa* Roxb. (*C. comosa*), a plant in Zingiberaceae family, has been widely used as a dietary supplement for relieving postmenopausal symptoms in Thailand [14]. Consistent with the presence of a phytoestrogen, hexane extract of *C. comosa* rhizomes prevent bone loss in estrogen deficient mice [15]. Diarylheptanoid, (3*R*)-1,7-diphenyl-(4*E*,6*E*)-4,6-heptadien-3-ol (hereafter DPHD), a novel phytoestrogen isolated from *C. comosa* [16] has several pharmacological properties including estrogenic-like activity [17], [18] and anti-inflammatory effects [19]. Recently, DPHD was found to activate Wnt/β-catenin signaling and promote mouse preosteoblastic (MC3T3-E1) cell proliferation through the estrogen receptor pathway [20]. Similarly, human osteoblast cell differentiation and function were also enhanced upon DPHD treatment [21] suggesting that DPHD may have a beneficial effect in preventing bone loss in patients experiencing estrogen deficiency.

The biological activities of DPHD appear to be selective with anabolic effects predominantly on osteoblasts. We hypothesized that DPHD may have a beneficial effect in preventing bone loss due to estrogen deficiency. In the present study, we investigated the bone sparing effect of DPHD in ovariectomized-rats that exhibit estrogen deficiency. The effect of DPHD on bone mineral density (BMD), changes to bone microarchitecture, and biochemical markers of bone turnover were determined after a 12-week course of treatment. Our analysis provides mechanistic insight into the beneficial effects of the phytoestrogen DPHD in reducing bone loss in estrogen deficient rats and suggests a potential clinical use for DPHD in menopausal women.

## Materials and Methods

The animal experimental protocol was approved by the committee on Animal Care and Use, Faculty of Science, Mahidol University (approval protocol number: MUSC-171). All animal experiments were performed in accordance with the guidelines of National Laboratory Animal Center, Mahidol University.

### Chemicals and Plant Materials

**Figure 1:**
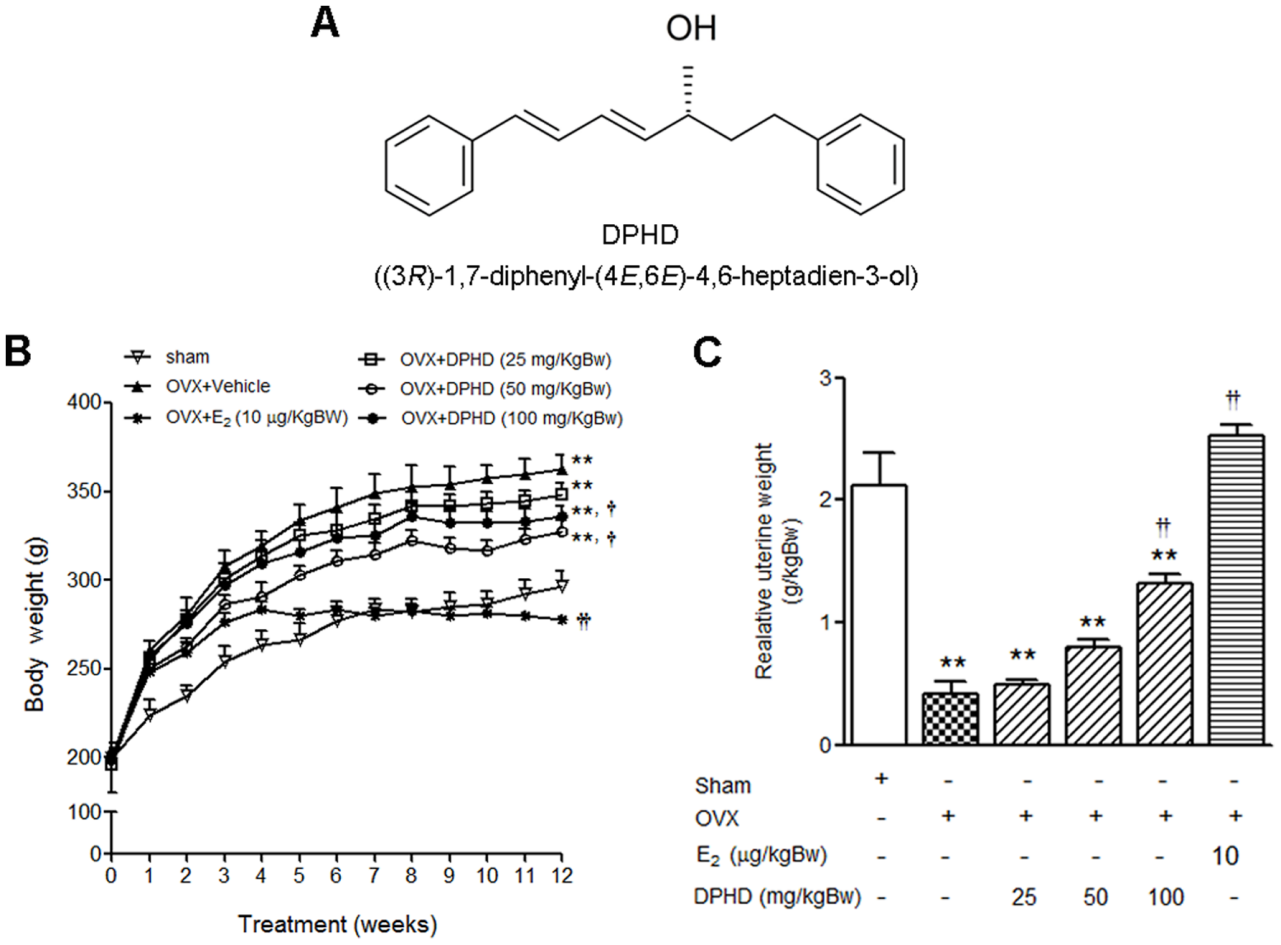
Estrogenic activity of DPHD compared to E_2_. Structure of the phytoestrogen diarylheptanoid DPHD, (3*R*)-1,7-diphenyl-(4*E*,6*E*)-4,6-heptadien-3-ol, isolated from the rhizome of *C. comosa* (A). Effects of DPHD on body weight (B) and uterine weight (C) of sham-operated and ovariectomized (OVX) rats receiving vehicle and various doses of DPHD (25, 50 and 100 mg/kg Bw) or 17β-estradiol (E_2_, 10 µg/kg Bw) for 12 weeks. Results are expressed as the mean ± SEM, n = 6–8. **p<0.01, significantly different from sham rats. ^†^p<0.05 and **^††^**p<0.01, significantly different from OVX rats.

Preparation of phytoestrogen diarylheptanoid (3*R*)-1,7-diphenyl-(4*E*,6*E*)-4,6-heptadien-3-ol (DPHD) from C. comosa was performed as previously described [16], [21]. Rhizomes of *C. comosa* were purchased from the Kampaengsaen district, Nakhon Pathom province, Thailand. No specific permission is required for these activities and the field study did not involve endangered or protected species. Briefly, rhizomes were cut into small pieces, dried and ground to powder then extracted with n-hexane in a Soxhlet extractor. After removal of the solvent *in vacuo*, a pale brown viscous oil was obtained. The DPHD was isolated from the hexane extract as a major component (23.9%) by repeated silica gel column chromatography. DPHD was eluted with hexane-dichloromethane and each step utilized an increasing quantity of the more polar solvent. The structure of DPHD was confirmed and the absolute stereochemistry at the 3-position was determined to be R by nuclear magnetic resonance and mass spectroscopy, the same as that of DPHD previously isolated [16]. The purity of the isolated material was assessed by TLC and NMR spectroscopy and estimated to be 99% pure. The chemical structure is shown in Figure 1A.

17β-estradiol (E_2_) and p-nitrophenyl phosphate were purchased from Sigma-Aldrich Chemical Company (MO, USA). Methyl methacrylate, 2-ethoxyethyl acetate and orange G were obtained from Merck Company (Darmstadt, Germany). Haematoxylin, fushin acid, and DePex mounting medium were purchased from VWR International Ltd. (Poole, England). All compounds were initially dissolved in 5% DMSO and diluted in olive oil to the final doses.

### Animals and Treatments

Eight-week-old female Sprague-Dawley rats, weighing 200–220 g, were supplied by the National Laboratory Animal Centre of Thailand (Salaya, Nakornpathom, Thailand). Animals were housed in standard stainless steel cages under controlled conditions: temperature at 25±2°C, relative humidity of 50–60%, a 12-h light/dark cycle, and allowed free access to food (rat pellets, C.P. rat feed, Pokphand Animal Fed Co. Ltd., Bangkok, Thailand) and water. Rats were randomly assigned to sham-operated control and ovariectomized (OVX) groups. In OVX animals, both sites of ovaries, which are the primary source of endogenous estrogen, were removed under general anesthesia using pentobarbital sodium (50 mg/kg Bw, i.p.). Animals were allowed to recover from surgery for one week prior to use in experiments. Rats were divided into six groups of six to eight animals each as follows: sham operated control receiving vehicle (olive oil); OVX rats receiving vehicle (olive oil, i.p.); OVX rats receiving DPHD at doses of 25, 50 and 100 mg/kg Bw (i.p.); OVX rats receiving 17β-estradiol (E_2_) at a dose of 10 µg/kg Bw (s.c.) as a positive control. DPDH and E_2_ were daily administered for 12 weeks and body weights were recorded weekly. All rats were given subcutaneous injections of 10 mg/kg calcein, a fluorochrome bone marker, on Day 7 and Day 1 before animals were sacrificed. At the end of treatments, animals were euthanized with an overdose of sodium pentobarbital. Serum was collected and stored at −70°C until use and the uterus was removed and weighed. Tibial bones were excised, kept in saline-soaked gauze, covered with plastic and stored at −20°C prior to analysis.

### Measurement of Bone Mineral Density (BMD)

The bone mineral density of left tibia was measured *ex vivo* by peripheral Quantitative Computed Tomography (pQCT; XCT Research SA^+^, Stratec Medizintechnik GmbH., Germany) according to a previously protocol [22]. In brief, both the trabecular and cortical bone density were scanned in cross-sectional plane at metaphyseal sites of tibias. Proximal tibial metaphysis was measured 2 mm below the growth plate. All bones were scanned at 0.5 mm intervals using a voxel size of 0.09 mm×0.09 mm×0.09 mm. The trabecular bone was determined using contour mode 2 and peel mode 2 with a threshold value of 720 mg/cm^3^. The cortical bone was determined using separation mode 2 with a threshold value of 900 mg/cm^3^. All parameters were analyzed using XCT-5.50E software (Stratec Medizintechnik GmbH., Germany).

### Bone Histomorphometric Analysis

All bone histomorphometries were conducted at the proximal metaphyseal region of the right tibia. The adhering tissues and bone marrow were removed from tibias followed by fixation for 3 days in 70% (vol/vol) ethanol, as previously described [23]. Bones were then dehydrated in 95, and 100% (vol/vol) ethanol for 3 and 2 days, respectively, followed by embedding and undecalcification in methyl methacrylate resin at 42°C for 48 h. To obtain 7 µm and 12 µm thick sections, the embedded tibia was cut in longitudinal section using a microtome (model RM2255; Leica, Nussloch, Germany). The region of tibial studied was the secondary spongiosa, the trabecular part of proximal tibia, at 1–2 mm distal to the epiphysial plate and extending to 6 mm. The 7 µm sections were deplasticified in 2-ethoxyethyl acetate and stained with Goldner’s trichrome then analyzed under bright field microscopy. The structural variables were examined using the histology section and parameters measured include trabecular bone volume, normalized by tissue volume (BV/TV, %), trabecular number (Tb.N, mm^−1^), trabecular thickness (Tb.Th, µm) and trabecular separation (Tb.Sp, µm). The 12 µm sections of proximal tibia were left unstained to determine the mineral apposition rate (MAR), an index of osteoblastic activity, calculated by dividing the mean distance between double labels of the calcein with time interval between the administration of the two labels. Bone formation rate (BFR/TV) is another dynamic parameter that is an index of bone turnover in general and bone formation in particular and allows for the determination of the age of bone [24]. All slides were analyzed under a light/fluorescent microscope using a computer assisted Osteomeasure system (Osteometric, Atlanta, GA), software version 4.1. Bone histomorphometric parameters were reported according to the American Society for Bone and Mineral Research Nomenclature Committee [25].

### Serum Bone Biomarkers Assay

Tartrate-resistant acid phosphatase (TRAP) activity, a bone resorption marker, was determined by using microplate assay method. 4-nitrophenyl phosphate (4-NPP) was used as the substrate according to the procedure of Lau *et al*. with modification [26]. Serum was incubated for 30 min at 37°C with a substrate solution consisting of 7.6 µmol/L 4-NPP in 100 µmol/L sodium acetate buffer containing 50 µmol/L sodium tartrate (pH 5.5). 1 µmol/L NaOH was added to stop the reaction and the absorbance at 405 nm was monitored to detect product formation. Serum osteocalcin concentration, a bone turnover marker, was measured using an enzyme immunoassay (EIA) kit specific for rat osteocalcin (Biomedical Technology, Staughton, IN, USA).

### Statistical Analysis

All data are expressed as means ± SEM and were analyzed using one-way analysis of variance (ANOVA) and Newman–Keuls post-hoc test using SPSS for Windows, Version 17.0 (Chicago, IL, USA). A non parametric Wilcoxon-type test for trend (Cuzick’s Test for Trend) was employed for evaluation of the trend across the groups. Differences were considered statistical significant at p<0.05.

## Results

### Effects of Ovariectomy and DPHD Treatment on Body Weight and Uterine Weight

All rats exhibited an increase in body weight during the 12 weeks of treatment, particularly in OVX rats. As shown in Figure 1B, at the end of experiment, the body weight gain was consistently highest in OVX control. However, the increases in body weights of OVX rats was suppressed by treatment with E_2_ (10 µg/kg Bw) to levels similar to the sham controls. Treatments of OVX rats with DPHD at doses of 50 and 100 mg/kg BW also significantly decreased body weight compared to OVX controls. However, the effect of DPHD on body weight was not as pronounced as that seen with E_2_ (Figure 1B). These results indicate that DPHD partially suppressed body weight gain in OVX rats. The uterine weights of OVX rats was also changed but in this case a significant decrease was observed when compared to sham controls (p<0.01). Uterine weight was increased in OVX rats following treatment with estrogen and DPHD, though a significant increase was only observed at 100 mg/kg Bw of DPHD (p<0.01) (Figure 1C).

### Effects of DPHD on *ex vivo* Bone Mineral Density (BMD)

**Figure 2:**
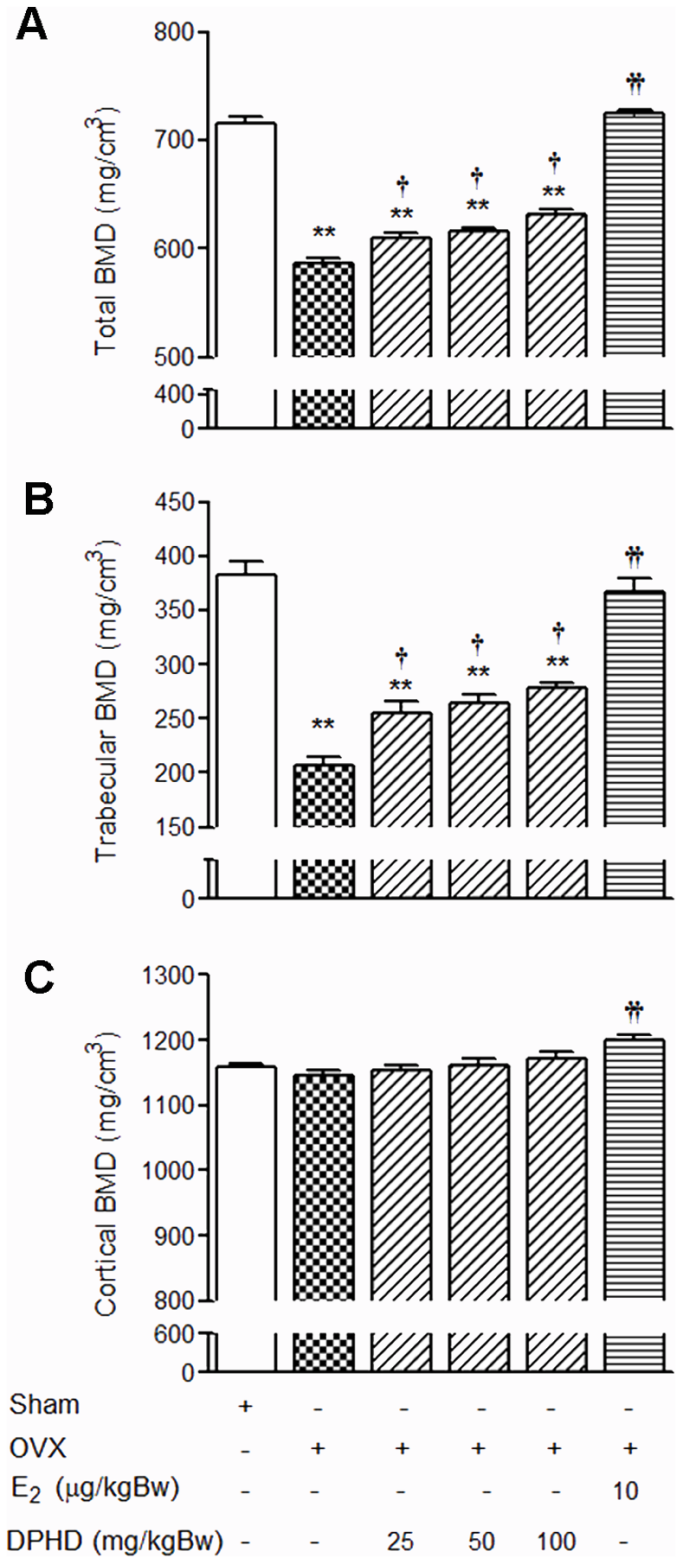
DPHD increases ex vivo bone mineral density (BMD), as measured by pQCT. Total (A), trabecular (B), and cortical (C) BMD of tibial metaphysis from sham-operated and ovariectomized (OVX) rats receiving vehicle, DPHD (25, 50 and 100 mg/kg Bw) or 17β-estradiol (E_2_, 10 µg/kg Bw) for 12 weeks. Results are expressed as mean ± SEM, n = 6−8. **p<0.01, significantly different from sham rats. ^†^p<0.05 and ^††^p<0.01, significantly different from OVX rats.

Both total and trabecular bone mineral density (BMD) of tibial metaphysis were markedly decreased in OVX rats (at 12 weeks) compared to those of sham controls (Figure 2A and 2B, respectively). E_2_ treatment (10 µg/kg Bw) effectively prevented the decreases in total and trabecular BMD. Treatments with DPHD at doses of 25, 50, and 100 mg/kg Bw also prevented the decrease in total and trabecular BMD compared to the OVX group given the vehicle control. Similar to the effect observed for body weight, treatment with DPDH did not restore BMD to the level seen in the sham-operated group. Interestingly, DPHD had no effect on the cortical BMD of tibial metaphysis though a protective effect was observed with E_2_ (Figure 2C). These findings suggest that DPHD predominantly only protects against trabecular bone loss, while E_2_ effectively prevents the loss of both trabecular and cortical bones.

### Effects of DPHD on Bone cross Sectional Area and Thickness

In Table 1, the total, trabecular and cortical bone cross sectional areas (CSA) of tibia are shown. In OVX rats, total and trabecular CSA of tibia were increased by 12% and 20%, respectively, compared to sham controls. Treatment with E_2_, and DPHD at doses of 50 and 100 mg/kg Bw prevented the increases in cross sectional area. However, there was no significant change in cortical area and thickness.

**Table 1:** Effect of DPHD on bone area and thickness of OVX rats.

Groups/Parameters	Total CSA (mm^2^)	Trebecular CSA (mm^2^)	Cortical CSA (mm^2^)	Cortical thickness (mm)
Sham	14.61±0.38	7.89±0.30	6.21±0.14	0.68±0.011
OVX + Vehicle	16.50±0.50*	9.51±0.49*	6.41±0.11	0.70±0.010
OVX + DPHD (25 mg/kg)	16.15±0.42*	9.03±0.40*	6.45±0.12	0.70±0.005
OVX + DPHD (50 mg/kg)	14.99±0.39†	8.36±0.15†	6.02±0.12	0.69±0.006
OVX + DPHD (100 mg/kg)	14.72±0.44†	8.12±0.39†	5.91±0.13	0.69±0.005
OVX + E_2_ (10 µg/kg)	14.73±0.48†	7.82±0.51†	5.97±0.10	0.68±0.007

Total, trabecular, and cortical cross sectional area (CSA) and cortical thickness were measured from sham-operated and ovariectomized (OVX) rats receiving vehicle, DPHD (25, 50 and 100 mg/kg Bw) or 17β-estradiol (E_2_, 10 µg/kg Bw) for 12 weeks. Data are expressed as mean ± SEM, n = 6−8.

* p<0.05, significantly different from sham rats.

† p<0.05, significantly different from OVX rats.

### Effects of DPHD on Trabecular Bone Microarchitectural Changes

**Figure 3:**
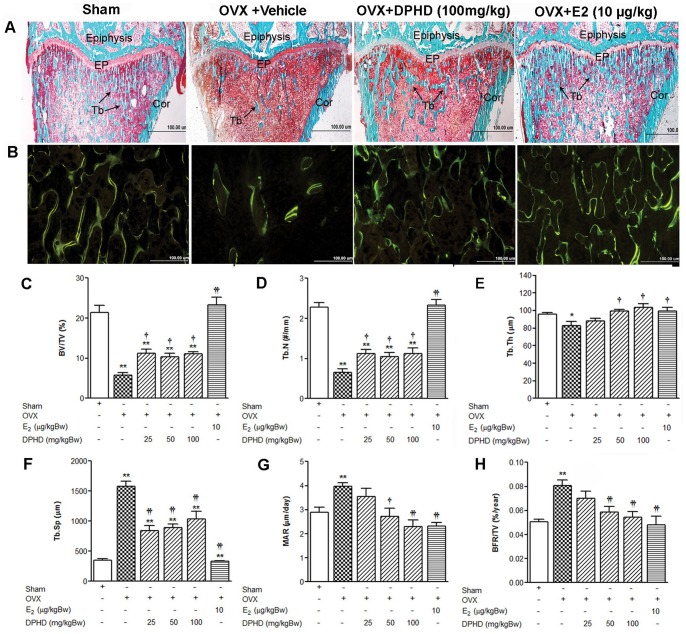
Reversal of OVX induced bone microarchitectural changes by DPHD treatment. Representative 2D images of the proximal tibial metaphysic (trabecular structure) of sham operated and OVX rats receiving vehicle, DPHD (DPHD 100 mg/kg Bw and 17β-estradiol (E_2_, 10 µg/kg Bw) for 12 weeks. Samples were stained with Goldner’s trichrome for bright-field microscopy at a magnification of 2X showing the following: epiphysis, epiphyseal plate (EP), trabecular bone (Tb), and cortical bone (Cor) (A). Fluorescence micrographs (calcein labeling) (B). Static parameters: trabecular bone volume normalized by tissue volume (BV/TV, %) (C), trabecular number (Tb.N, mm^−1^) (D), trabecular thickness (Tb.Th, µm) (E), and trabecular separation (Tb.Sp, µm) (F). Dynamic parameters: mineral apposition rate (MAR) (G) and bone formation rate (BFR) (H). Data are expressed as the mean ± SEM, n = 6−8. **p<0.01 significantly different from sham rats and ^†^p<0.05 and ^††^p<0.01, significantly different from OVX rats.

Both static and dynamic changes in histomorphometry of the proximal tibial metaphysis were evaluated. The growth plate and spongiosa region of the proximal tibia of sham, OVX, OVX+DPHD (100 mg/kg Bw), and OVX+E_2_ (10 µg/kg Bw) rats are shown in Figure 3A. Compared to the sham rats, a decrease in trabecular bone and connectivity was observed in OVX rats indicating that ovariectomy resulted in the deterioration of trabecular bone microstructure. However, treatment with E_2_ completely protected against this deterioration with partial protection observed with DPHD treatment. Ovariectomy also induced a marked decrease in the trabecular bone volume (BV/TV) compared to that of the sham rats (73% reduction) (Figure 3C) and again treatment with E_2_ completely restored trabecular bone volume to levels seen in the sham controls. All doses of DPHD significantly increased BV/TV (Figure 3C) and trabecular number (Tb.N) (p<0.05) (Figure 3D) in OVX rats but these values were reduced compared to the sham and E_2_ treated animals. DPHD treatment also increased trabecular thickness (Tb.Th) in OVX rats but significant difference was not observed at low dose of DPHD (25 mg/Kg Bw)-treated group (Figure 3E). Trabecular separation (Tb.Sp), another important structural index for static micro-structural changes of bone, was markedly increased in OVX rats compared to sham controls. E_2_ treatment was capable of significantly decreasing the separation of bone to the level seen in the sham controls. The Tb.Sp in animals treated with DPHD was also significantly reduced but were significantly higher than that for the sham control group (Figure 3F). These results suggest that DPHD treatment improved the connectivity of trabecular bone in the ovariectomized rats though to a lesser degree than treatment with E_2_.

The dynamic bone histomophometry was assessed using fluorescence microscopy to monitor the uptake of calcein, a fluorochrome bone marker (Figure 3B). Bone formation and mineralization, expressed as mineral apposition rate (MAR), were determined by the distance between two fluorochrome markers given at different days and divided by the number of days between administrations. This index reflects the activity of osteoblasts. Compared to sham animals, MAR in OVX rats was significantly increased from 2.89±0.2 to 3.98±0.1, indicating that ovariectomy caused an increase in new bone formation leading to increase bone turnover (Figure 3G).

Treatments with E_2_ or DPHD at doses of 50, and 100 mg/kg BW significantly decreased MAR to levels seen in the sham controls. Bone formation rate (BFR), an index of bone turnover provides the best correlation with the serum bone turnover markers [24], and the bone formation rate per total volume (BFR/TV) was significantly increased after ovariectomy (Figure 3H). Similar to MAR, the increase in BFR/TV in OVX animals was effectively prevented by treatment with either E_2_ or DPHD. The reduction of MAR and BFR in DPHD treated rats indicated that DPHD was capable of decreasing bone turnover rate in a similar manner as E_2_.

### Effects of DPHD Treatment on Biochemical Bone Turnover Markers

**Figure 4:**
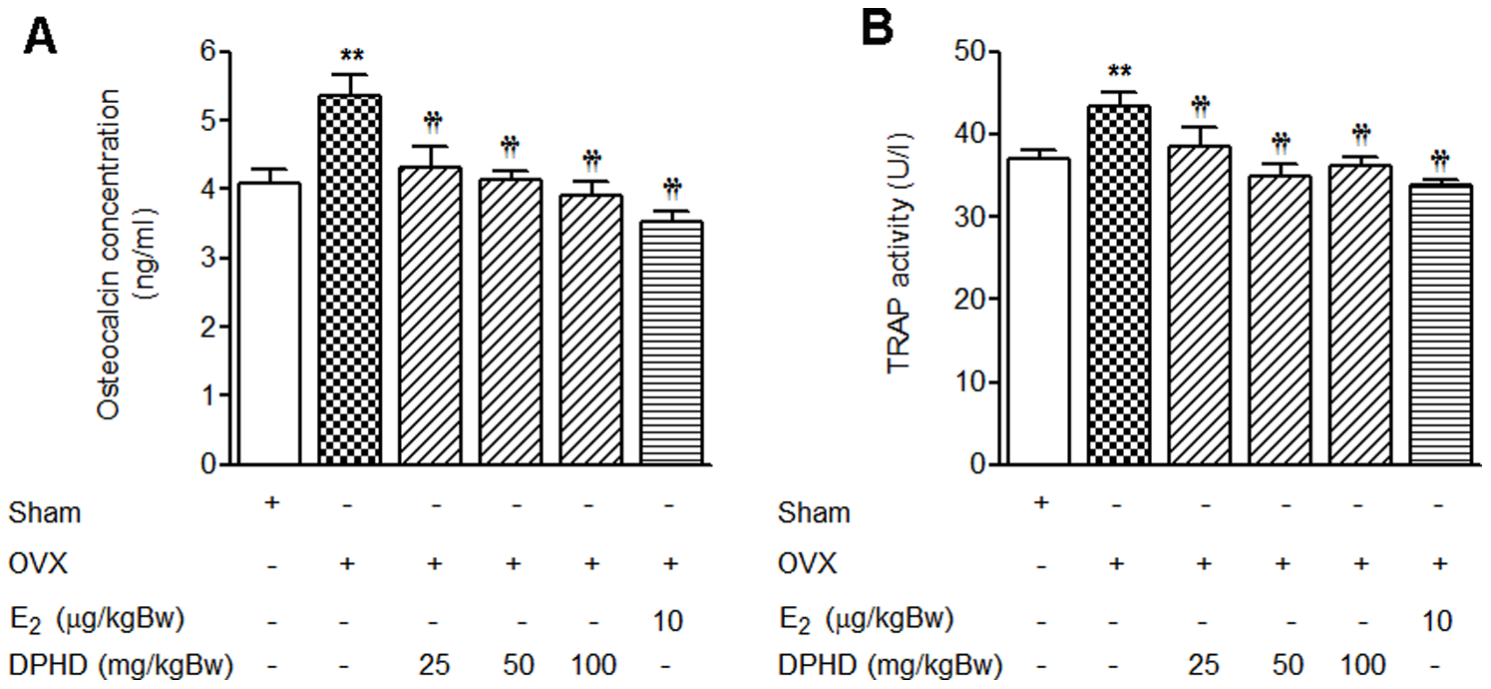
Effects of DPHD on biochemical bone turnover markers. Serum osteocalcin levels (A) and TRAP activity (B) of sham-operated and ovariectomized (OVX) rats receiving the indicated doses of DPHD (25, 50 and 100 mg/kg Bw) or 17β-estradiol (E_2_, 10 µg/kg Bw) for 12 weeks. Results are expressed as the mean ± SEM, n = 6−8. **p<0.01 significantly different from sham rats and ^†^p<0.05 and ^††^p<0.01, significantly different from OVX rats.

To evaluate the effect of E_2_ and DPHD treatments on bone turnover in OVX rats, we measured the serum osteocalcin concentration and tartrate-resistant acid phosphatase activity. As shown in Figure 4A, the serum osteocalcin concentration in OVX rats was significantly higher than that in sham animals and DPHD treatment of OVX rats significantly reduced the serum osteocalcin concentration. These results indicate that DPHD prevents the ovariectomy-induced increase of bone turnover in rats. The TRAP activity of osteoclast, an index of bone resorption, was 25% higher in OVX rats compared to the sham group and DPHD treatment restored TRAP activity to level similar to those of Sham and E_2_-treated groups (Figure 4B). Since the decreases in bone turnover and resorption markers are related to the suppression of bone formation rate, these results suggest that DPHD decreased the bone turnover rate by suppressing osteoclast activity in OVX rats.

## Discussion

The present study has demonstrated for the first time of the bone sparing effect of a novel diarylheptanoid phytoestrogen (DPHD) isolated from *C. comosa*. In ovariectomy-induced osteopenia (OVX), a deterioration in trabecular bone microarchitecture (12 weeks after ovariectomy) clearly led to the loss of bone mass in rats. Treatments with DPHD effectively prevented the trabecular bone loss and improved bone microstructure. Moreover, markers of bone turnover, including osteocalcin and TRAP activity, were decreased in DPHD treated animals. These results suggest that DPHD provides a protective effect against OVX-induced bone loss that is associated with decreased bone turnover through suppressing bone resorption.

The integrity of skeletal is maintained through a bone remodeling process that balances bone formation and bone resorption [4] and estrogen plays an important role in the maintenance of bone mass [27]. The rapid decline of estrogens in postmenopausal women results in an imbalance in the bone remodeling process leading to osteoporosis [28]. Mornitoring of BMD is important for diagnosis and the treatment of osteoporosis as decreased bone mass is a major characteristic of this disease. In this study, decreased BMD in OVX rats, determined using pQCT was observed only in the metaphysic of the tibia, which has a greater proportion of trabecular bone in the proximal end. Trabecular bone, a sponge-like bone found at the ends of long bones and vertebrae, contains osteoblasts and osteoclast on its surface and is more active in bone turnover and bone remodeling compared to cortical bone [29], [30]. Indeed, our analysis of OVX rats showed only loss of trabecular BMD. This finding is consistent with previous studies that report the loss of bone in adult OVX rats was more prominent in trabecular than cortical bone [31]. However the loss of trabecular BMD in OVX rats was attenuated by DPHD treatment. Consistent with reports that estrogen decreases periosteal bone formation and radial growth [32], OVX rats displayed an increase in cross sectional bone area indicating that radial growth was increased. Similar to estrogen, treatment with DPHD prevent the increase in bone area in OVX animals. The improvement in bone measurements following DPHD treatment may be partly attributed to its estrogenic like activity, as evidenced by increased uterine weight in DPHD exposed animals (Figure 1C) and our earlier study on uterotropic activity of DPHD [17], [18].

A rapid reduction in trabecular bone volume is known to occur following ovariectomy and is associated with an increase in bone turnover rate resulting from an excessive osteoclast activity [33]. Our analysis of the destruction of bone microarchitecture, another important characteristic of osteoporosis, evaluated using bone histomorphometry is consistent with an increased rate of bone turnover in OVX rats. Ovariectomy also markedly decreased static indices, including trabecular bone volume, thickness, and number with an increase in trabecular separation. In addition to direct effects on bone morphology, monitoring changes in circulating bone biochemical markers can also reveal the status of bone remodeling process [34]. These markers include osteocalcin, an osteoblast-specific bone formation marker, and tartrate-resistant acid phosphatase (TRAP) activity, an osteoclast-specific bone resorption marker [34], [35]. A dramatic increase in serum osteocalcin and TRAP activity was observed 12 weeks following ovariectomy, confirming that bone loss was due to an increase in bone turnover rate. DPHD reduced these markers of bone turnover in OVX rats suggesting that DPHD prevented trabecular bone loss and micro-architecture deterioration by suppressing the rate of bone turnover either by decreasing bone resorption or increasing bone formation.

DPHD at doses of 25 and 50 mg/kg BW preserved bone mass in OVX rats without showing an uterotrophic effect. These results indicate that the beneficial effect of DPHD on bone is not limited to its estrogenic property but also mediate through other biological effects of DPHD, such as an anti-inflammatory activity [19]. Inflammation is one of the causal factors of osteoporosis and several cytokines, such as IL-1, M-CSF and RANKL, are involved in the pathogenesis of osteoporosis. The role of these cytokines is to activate osteoclast differentiation and bone resorption [36]. RANKL, a TNF family member, is synthesized by the osteoblast and is an essential cytokines for activation of osteoclast formation, function, and survival [37]. The interaction of RANKL and RANK stimulates the osteoclastogenesis, the coupling process between the osteoblast and osteoclast to control bone remodeling [38]. Inhibiting the interaction of RANKL and RANK may have benefits in the treatment of osteoporosis and DPHD treatment reduces mRNA level of RANKL produced by osteoblast cells during differentiation [21]. The inhibitory effect of DPHD on RANKL may in turn attenuate the interaction of RANKL and RANK and subsequently reduce the downstream inflammatory cytokine induced osteoclastogenesis and bone resorption. Estrogen deficiency in OVX rats is associated with the local disturbance of cytokines in bone marrow, leading to an increase in osteoclast numbers that ultimately penetrate trabecular bone and cause deep resorption cavities [39]. Consequentially, trabecular bones are lost and the remaining bones are less dense, thinner, and widely separated [40]. Changes in cytokine levels in OVX rats may be attenuated by DPHD treatment. If this is the case, then it suggests that DPHD may suppress osteoclast activity. The inhibitory effect of DPHD on both RANKL mRNA expression and interaction of RANKL and RANK in osteoblast cells may in part account for attenuation of bone turnover and preserving bone mass after ovariectomy [21]. Pharmacokinetic analysis indicates that the amount of DPHD used in treatment of OVX rats in the present study would provide an effective concentration in the range similar to that reported in the *in vitro* study [21], [40]. However, the response of cytokines to DPHD treatment in OVX rats has not been investigated and any effect of DPHD on osteoclast cells and the inflammatory system remains to be elucidated.

In conclusion, this is the first report on the effect of DPHD on bone turnover and protection of trabecular bone loss in OVX rats. Our results indicate that the novel phytoestrogen, DPHD, exhibits low uterotrophic activity and has potential in clinical applications for preserving bone mass and structure in postmenopausal osteoporosis.
